# Staged or simultaneous operations for ventriculoperitoneal shunt and cranioplasty: Evidence from a meta‐analysis

**DOI:** 10.1111/cns.14347

**Published:** 2023-07-12

**Authors:** Jun Zhang, Xinyu Deng, Qiang Yuan, Pengfei Fu, Meihua Wang, Gang Wu, Lei Yang, Cong Yuan, Zhuoying Du, Jin Hu

**Affiliations:** ^1^ Department of Neurosurgery, Huashan Hospital, Shanghai Medical College Fudan University Shanghai China; ^2^ National Center for Neurological Disorders Shanghai China; ^3^ Shanghai Key Laboratory of Brain Function and Restoration and Neural Regeneration Shanghai China; ^4^ Neurosurgical Institute of Fudan University Shanghai China; ^5^ Shanghai Clinical Medical Center of Neurosurgery Shanghai China; ^6^ Department of Neurosurgery & Neurocritical Care, Huashan Hospital, Shanghai Medical College Fudan University Shanghai China

**Keywords:** complications, craniectomy, cranioplasty, hydrocephalus, ventriculoperitoneal shunt

## Abstract

**Objective:**

To date, there is no consensus on the surgery strategies of cranioplasty (CP) and ventriculoperitoneal shunt (VPS) placement. This meta‐analysis aimed to investigate the safety of staged and simultaneous operation in patients with comorbid cranial defects with hydrocephalus to inform future surgery protocols.

**Methods:**

A meta‐analysis of PubMed, Ovid, Web of Science, and Cochrane Library databases from the inception dates to February 8, 2023 adherent to PRISMA guidelines was conducted. The pooled analyses were conducted using RevMan 5.3 software. The outcomes included postoperative infection, reoperation, shunt obstruction, hematoma, and subdural effusion.

**Results:**

Of the 956 studies initially retrieved, 10 articles encompassing 515 patients were included. Among the total patients, 193 (37.48%) and 322 (62.52%), respectively, underwent simultaneous and staged surgeries. The finding of pooled analysis indicated that staged surgery was associated with lower rate of subdural effusion (14% in the simultaneous groups vs. 5.4% in the staged groups; OR = 2.39, 95% CI: 1.04–5.49, *p* = 0.04). However, there were no significant differences in overall infection (OR = 1.92, 95% CI: 0.74–4.97, *p* = 0.18), central nervous system infection (OR = 1.50, 95% CI: 0.68–3.31, *p* = 0.31), cranioplasty infection (OR = 1.58, 95% CI: 0.50–5.00, *p* = 0.44), shunt infection (OR = 1.30, 95% CI: 0.38–4.52, *p* = 0.67), reoperation (OR = 1.51, 95% CI: 0.38–6.00, *p* = 0.55), shunt obstruction (OR = 0.73, 95% CI: 0.25–2.16, *p* = 0.57), epidural hematoma (OR = 2.20, 95% CI: 0.62–7.86, *p* = 0.22), subdural hematoma (OR = 1.20, 95% CI: 0.10–14.19, *p* = 0.88), and intracranial hematoma (OR = 1.31, 95% CI: 0.42–4.07, *p* = 0.64). Moreover, subgroup analysis failed to yield new insights.

**Conclusions:**

Staged surgery is associated with a lower rate of postoperative subdural effusion. However, from the evidence of sensitivity analysis, this result is not stable. Therefore, our conclusion should be viewed with caution, and neurosurgeons in practice should make individualized decisions based on each patient's condition and cerebrospinal fluid tap test.

## Graphical abstract

Up to now, there is no consensus on the surgery strategies of cranioplasty and ventriculoperitoneal shunt placement in patients with co‐morbid cranial defects with hydrocephalus. Therefore, a meta‐analysis was conducted to investigate this issue. Our cautiously indicated that patients undergo staged cranioplasty and ventriculoperitoneal shunt procedures might benefit from a potentially lower trend in subdural effusion rate.

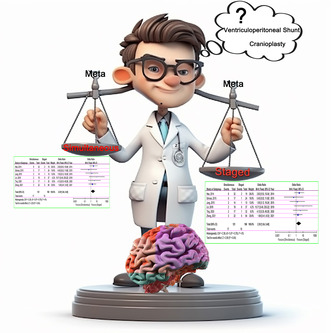

## 1 INTRODUCTION

Decompressive craniectomy (DC) is an ancient surgical procedure, dating back to trepanation around 12,000 BC.^1^ It is generally believed that the earliest report in modern neurosurgery using DC to treat neurosurgical disorders was Kocher's treatment of patients with elevated intracranial pressure (ICP) after traumatic brain injury (TBI) in 1901.^2^ DC is mainly used as a second‐line treatment in patients with drug‐refractory intracranial hypertension caused by various reasons, including TBI, intracerebral hemorrhage (ICH), subarachnoid hemorrhage (SAH), and ischemic stroke (IS), etc.^3^, ^4^, ^5^, ^6^, ^7^, ^8^

The disruption of cerebral blood flow and cerebrospinal fluid (CSF) hydrodynamics after DC puts patients at higher risk of subdural effusion and hydrocephalus, potentially resulting in worse neurological outcomes.^9^, ^10^, ^11^ The sinking of the scalp due to the lack of skeletal support affects the blood supply, neurological function of the cortex, and aesthetics, which may cause sinking skin flap syndrome (SSFS) with a series of neuropsychiatric symptoms.^12^, ^13^, ^14^ Therefore, cranioplasty (CP) is necessary in order to repair the cranial bone defect. In addition to mechanical protection and cosmetology, CP can also effectively improve cerebral blood flow and CSF hydrodynamics, thereby improving cerebral metabolism and promoting neurological function recovery.^15^, ^16^, ^17^, ^18^, ^19^, ^20^, ^21^

Due to the changes of CSF dynamics caused by DC, hydrocephalus is an important complication after DC, with an incidence rate of 10%–40%.^22^, ^23^, ^24^ Initial disease can also cause post‐DC hydrocephalus, such as hydrocephalus following TBI or spontaneous SAH. Hydrocephalus is a dilatation of the ventricular system of the brain accompanied by an altered intraventricular pressure, which leads to secondary brain tissue damage and affects neurological recovery.^25^ CSF shunt is the standard treatment for hydrocephalus, the most common of which is ventriculoperitoneal shunt (VPS), which has been reported to be used in approximately 5%–15% of patients after DC.^26^, ^27^

Clinicians have primarily focused on the optimal surgical protocol of CP and VPS placement for post‐DC hydrocephalus patients. However, there is currently no consensus on the staged or simultaneous of CP and VPS placement. In such circumstances, the formulation of the surgical plans will be interfered with by the neurosurgeon's personal preference. This preference has an appropriately subjective character, which is primarily reflected in the surgeon's potential perceived merits or demerits of staged or simultaneous operations. Recent studies suggested that simultaneous surgery had higher rate of postoperative complications, especially infections, compared with staged surgery.^28^, ^29^, ^30^, ^31^ However, some neurosurgery experts indicated that simultaneous CP and VPS placement may be reduce surgical procedures, potential risks associated with general anesthesia, length of stay, medical expenses, and several clinical studies also indicated that there were no significant difference in the incidence of postoperative adverse events between staged and simultaneous surgery.^32^, ^33^, ^34^, ^35^, ^36^, ^37^

Given the preceding facts, we conducted a meta‐analysis on the clinical research data published on CP and VPS placement in patients with cranial defect comorbid with hydrocephalus and the aimed to investigate the ideal surgical protocol (staged or simultaneous) by comparing the incidence of postoperative complications, including infection, reoperation, shunt obstruction, hematoma, and subdural effusion. Furthermore, if staged surgery is listed as overwhelming, then the order of CP versus VPS also needs to be clarified. Therefore, subgroup analysis of staged procedures was also put on the agenda.

## 2 METHODS

### 2.1 Search strategy

This systemic review and meta‐analysis was conducted according to the Preferred Reporting Items for Systematic Reviews and Meta‐Analyses (PRISMA) guidelines.^38^ The electronic database including PubMed, Ovid, Web of Science, and Cochrane Library were searched from the date of inception to February 8, 2023. A deliberately simple strategy was performed to cast a broad search.^39^ For this purpose, the keywords “cranioplasty” and “ventriculoperitoneal shunt” were applied to identify all relevant studies. The literature search was independently conducted by J.Z. and X.Y.D. to ensure the accuracy. Any discrepancy was solved by consultation of an investigator, not involved in the initial procedure. The reference lists were also screened to avoid missing any articles.

### 2.2 Inclusion and exclusion criteria

The inclusion criteria were as follows: (1) studies enrolling patients with hydrocephalus after DC (that is, comorbid cranial defect with hydrocephalus), (2) studies that involved both simultaneous and staged CP and VPS placement, (3) studies were divided into simultaneous and staged groups, (4) studies that clearly reported the data of adverse events, (5) prospective or retrospective studies. The exclusion criteria were as follows: (1) case reports, reviews, conference abstracts, and editorial letters; (2) studies that included patients who underwent other shunting operations (e.g., lumbar‐peritoneal shunt replacement); (3) studies lacking sufficient data.

### 2.3 Quality assessment

Quality assessment was independently conducted by two authors (Z.J. and X.Y.D.). The Newcastle‐Ottawa Scale (NOS) was used to evaluate the quality of the finally included studies. The NOS includes three aspects [selection, comparability, and exposure (case–control studies) or outcome (cohort studies)] and a total score of 9 points (0 = worst to 9 = best, ≥7 was defined as high quality).^40^

### 2.4 Data extraction

Two authors (J.Z. and X.Y.D.) reviewed every eligible study and extracted the data including (1) information of the literature (title, author, publication date, etc.); (2) features of the studies (design, inclusion criteria, staging order, sample sizes, etc.); (3) baseline characteristics (demographic characteristics, clinical features, etc.); and (4) data on adverse events, including infection, reoperation, shunt obstruction, hematoma, and subdural effusion. For studies with insufficient information, the reviewers contacted the primary authors, when possible, to acquire and verify the data. In the event of disagreements about the quality of the studies or the data extracted, a third reviewer's opinion was sought.

### 2.5 Statistical analysis

Data synthesis and analysis was used Review Manager (version 5.3, Cochrane Collaboration). The odds ratios (ORs) with 95% confidence intervals (CIs) was applied as the effect indicator for the dichotomous variables. We calculated 95% CIs performing the Mantel–Haenszel statistical method. *I*‐square (*I* ^2^) statistics and *Q* tests were applied to evaluate the impact of study heterogeneity on the findings of this meta‐analysis. According to the Cochrane review guidelines,^41^ if severe heterogeneity was present at *p* < 0.1 or *I* ^2^ > 50%, the randomized effect models was applied; otherwise, the fixed‐effect models was used. Subgroup analyses were performed according to the staged procedures (CP after VPS, CP after VPS, both, and unknown). Sensitivity analysis was performed by removing single study sequentially. Forest plots were charted for pooled results, and funnel plots were used if no less than five studies were included to assess the publication bias.

## 3 RESULTS

### 3.1 Search results

**FIGURE 1:**
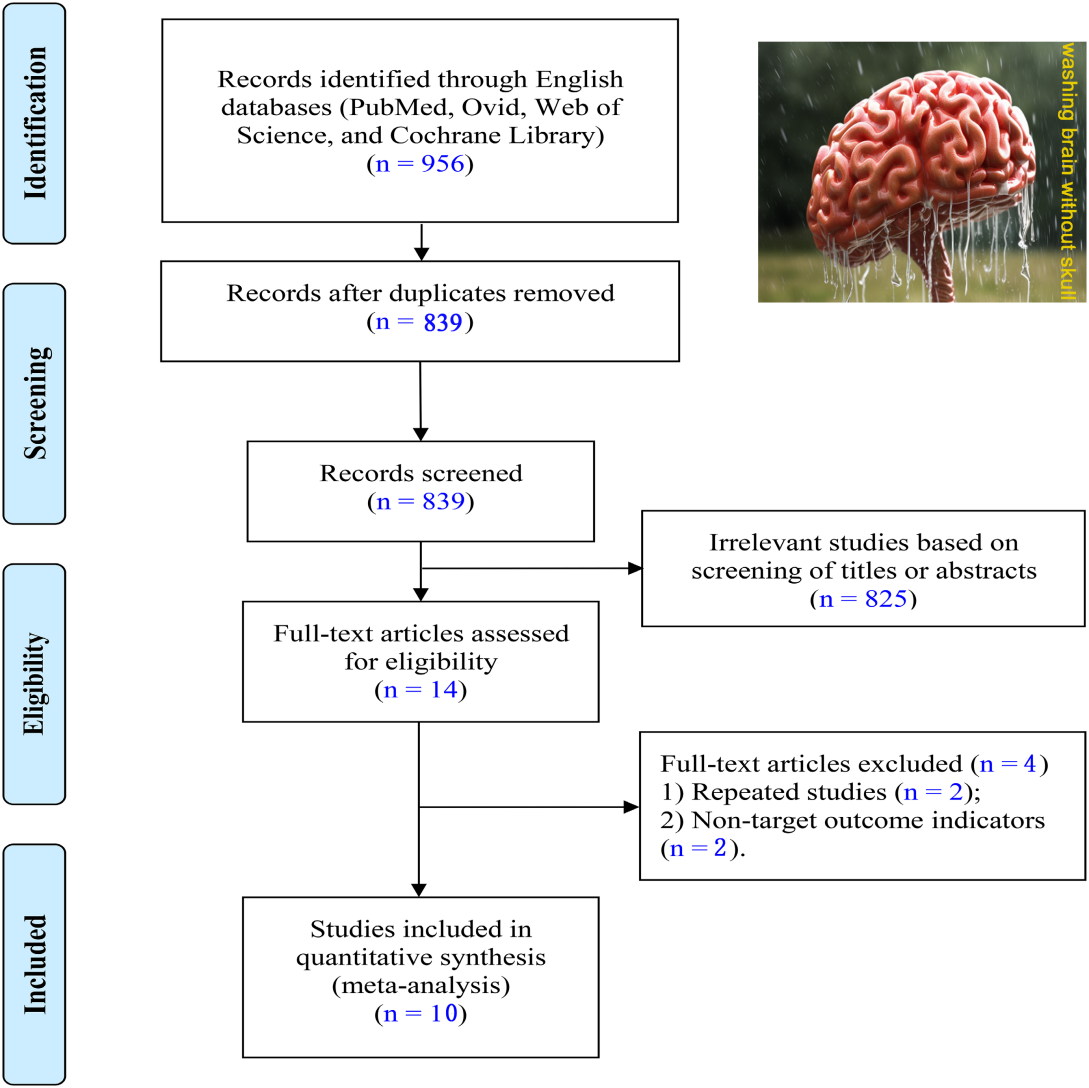
Flow diagram for literature search and screening process.

Our search yielded a total of 956 relevant records from various databases (PubMed, Ovid, Web of Science, and Cochrane Library). We excluded 117 duplicate articles, and selected 14 among the remaining 839 papers for full‐text assessment after screening titles and abstracts (Figure 1). The two repeated studies and two studies with non‐target outcome indicators were excluded. Finally, 10 articles met our inclusion criteria and were included in the pooled analysis.^28^, ^29^, ^30^, ^31^, ^32^, ^33^, ^34^, ^35^, ^36^, ^37^ These articles provided data on patient recruitment criteria and outcome variables, allowing us to conduct a thorough analysis of the available evidence. In addition, we performed a manual search of the reference lists of the included studies to identify any other potentially relevant articles.

### 3.2 Characteristics and quality

The baseline characteristics, quality, patient recruitment criteria, and outcomes of the 10 individual studies are summarized in Tables 1 and 2. All these studies were retrospective study and year of publication ranged from 2014 to 2021. A total of 515 patients underwent simultaneous or staged CP and VPS after DC. Among the total patients, 193 (37.48%) and 322 (62.52%) respectively underwent simultaneous and staged surgeries. Nine articles showed the order of CP and VPS in case of staged groups, and three studies were CP after VPS, two papers were VPS after CP, and four studies had both. The findings of quality evaluation demonstrated that the nine studies were of moderate quality (NOS score 5 or 6) and the one article was of low quality (NOS score 3).

**TABLE 1:** Baseline characteristics and quality of included studies.

Study & Year	Design	Age (year)	Number	Initial presentation	Staging order	Quality
		simultaneous, staged	Simultaneous, staged			
Gill, 2021	RS	60.9 ± 15.3, 62.5 ± 12.4	18, 63	TBI, SAH, ICH, IS, AVM	VPS after CP, CP after VPS	★★★★★★
Ting, 2020	RS	57.4 ± 13.7, 51.2 ± 17.5	17, 32	TBI	CP after VPS	★★★★★★
Zhang, 2021	RS	43.0–57.0, 33.0–53.0	22, 64	TBI, SAH, ICH	VPS after CP, CP after VPS	★★★★★★
Jung, 2015	RS	22–74	14, 10	TBI, ICH	VPS after CP	★★★★★
Lin, 2019	RS	57.5 ± 18.0, 52.6 ± 15.6	19, 37	TBI, ICH, SAH, IS, SDH	VPS after CP, CP after VPS	★★★★★★
Heo, 2014	RS	57.3, 55.3	32, 19	TBI, ICH, SAH, IS, BT	‐	★★★★★
Rosinski, 2020	RS	54.7 ± 10.1, 45.0 ± 10.6	18, 22	ICH, IS	CP after VPS	★★★★★
Schuss, 2015	RS	52.0 ± 13.0, 53.0 ± 18.0	17, 24	TBI, ICH, SAH, IS	CP after VPS	★★★★★★
Brelie, 2016	RS	42.8 ± 17.89	10, 27	TBI, IS	VPS after CP	★★★
Meyer, 2017	RS	43.0 ± 15.0	26, 24	TBI, ICH, SAH, IS, BT, BA	VPS after CP, CP after VPS	★★★★★

Abbreviations: AVM, arteriovenous malformation; BA, brain abscess; BT, brain tumor; CP, cranioplasty; ICH, intracerebral hemorrhage; IS, ischemic stroke; RS, retrospective study; SAH, subarachnoid hemorrhage; SDH, subdural hemorrhage; TBI, traumatic brain injury; VPS, ventriculoperitoneal shunt.

**TABLE 2:** Detailed inclusion and outcomes for each study.

Study & Year	Inclusion criteria	Outcomes
Gill, 2021	Patients underwent CP and VPS in simultaneous or staged operations following DC	Brain abscess, infections, intracranial hemorrhage, pneumocephalus, and neurological functional
Ting, 2020	Patients with TBI who had Glasgow Coma Scale score of <13 on admission and underwent unilateral DC	Infections, subdural hygroma, intracranial hematoma, reoperation, and neurological functional
	Patients underwent CP and VPS within 6 months after DC	
Zhang, 2021	Patients developed communicating hydrocephalus after DC and subsequently underwent CP and VPS placement	Infections, shunt malfunction, seizure, intracranial hematoma, subdural hygroma, and paradoxical herniation
	Patients who were not lost to follow‐up within 3 months	
Jung, 2015	Patients underwent DC, due to refractory intracranial hypertension after they had suffered a TBI or a vascular lesion	Intracranial hematoma, pseudomembranous colitis, subdural hygroma, infections, shunt malfunction, sunken bone plate
	All patients underwent early CP (an autologous bone flap, 5 to 8 weeks after DC)	
	Programmable shunt valve type (Codman‐Medos programmable VPS, Medos SA, Le Loche)	
Lin, 2019	Patients >18‐year‐old	Infections, over‐drainage, and reoperation
	Patients followed up for >3 months	
	Patients with non‐malignant brain tumor as the reason for DC	
Heo, 2014	Patients underwent CP and VPS operations after a DC for refractory intracranial hypertension	Intracranial hematoma, infections, and subdural hygroma
	The interval between the CP and VPS placement was within 6‐month	
	In all CP procedures were used autologous bone	
Rosinski, 2020	Adult patients who had undergone CP and VPS placement at any time after DC	Intracranial hematoma, reoperation, hospital‐acquired infection, cerebrospinal fluid leak, infections, shunt issues, length of stay
	Non‐pregnant	
Schuss, 2015	CP procedures with simultaneous or subsequent VPS placement in patients who previously underwent DC	Intracranial hematoma, infections, and subdural hygroma
	CP and VPS varied according to the treating neurosurgeon (no time limit)	
Brelie, 2016	Only patients with cranial vault Reconstruction after DC due to TBI and ischemic/hemorrhagic stroke	Infections, reoperation, subdural empyema, aseptic bone flap necrosis, neurological functional
	Patients were surgically treated in tertiary care center	
Meyer, 2017	All adult patients who underwent CP and VPS placement for any indication	Infections, shunt issues
	Follow‐up >3 months	

Abbreviations: CP, cranioplasty; DC, decompressive craniectomy; TBI, traumatic brain injury; VPS, ventriculoperitoneal shunt.

### 3.3 Risk of infection

**FIGURE 2:**
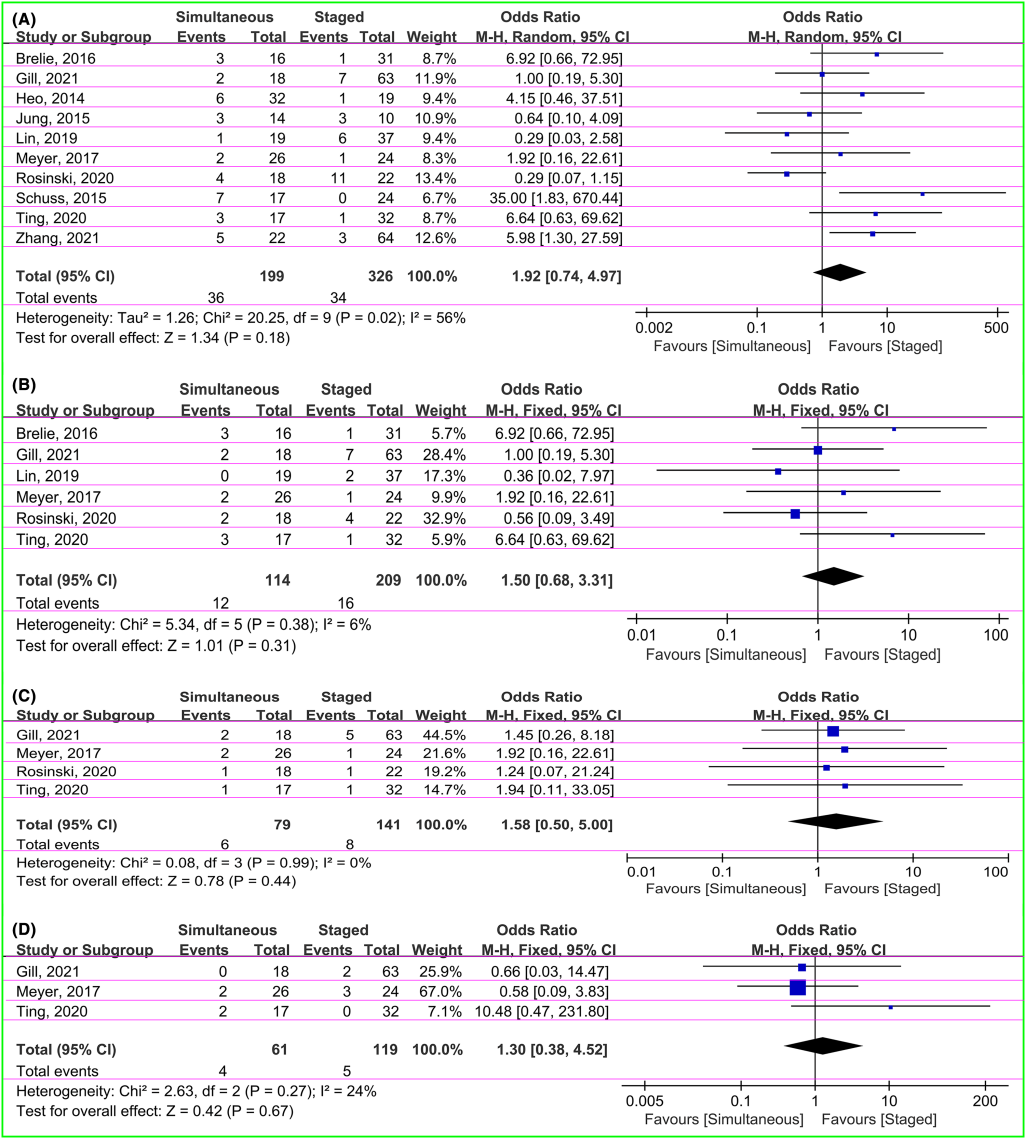
Forest plot showing comparison of overall infection (A), central nervous system infection (B), cranioplasty infection (C), and shunt infection (D) between simultaneous and staged groups.

The 10 papers were all included, with a total of 70 infected patients of all participants, including 36 (18.1%) in the simultaneous groups and 34 (10.4%) in the staged groups.^28^, ^29^, ^30^, ^31^, ^32^, ^33^, ^34^, ^35^, ^36^, ^37^ We applied OR to estimate the incidence of overall infection in both groups. The heterogeneity test suggested moderate difference between studies (χ^2^ = 20.25, *I* ^2^ = 56%, *p* = 0.02), so the fixed‐effect model was used. The pooled result revealed that although the incidence of infection was a trend toward greater in the simultaneous group, there were no statistically significant between two groups (OR = 1.92, 95% CI: 0.74–4.97, *p* = 0.18; Figure 2A).

The group‐level differences of the infection types were performed using OR to estimate the effect sizes. There were no substantial differences between studies (*I* ^2^ = 6%, 0%, and 24% on central nervous system infection, cranioplasty infection, and shunt infection, respectively), so the fixed‐effect model were applied. The central nervous system infection was reported in 28 of 323 patients in six articles, including 12 (10.5%) in the simultaneous groups and 16 (7.7%) in the staged groups (OR = 1.50, 95% CI: 0.68–3.31, *p* = 0.31; Figure 2B).^28^, ^32^, ^34^, ^35^, ^36^, ^37^ The cranioplasty infection was presented in 14 (six in the simultaneous groups and eight in the staged groups) of 220 patients in four studies (OR = 1.58, 95% CI: 0.50–5.00, *p* = 0.44; Figure 2C).^28^, ^32^, ^35^, ^37^ There were three papers showing the data of shunt infection (four in the simultaneous groups and five in the staged groups). The pooled OR was 1.30 (95% CI: 0.38–4.52, *p* = 0.67; Figure 2D).^28^, ^32^, ^37^ The findings suggested that the simultaneous groups had a trend of higher incidence of all infection types than the staged groups. However, there were no statistically significant difference between two groups.

### 3.4 Risk of reoperation

**FIGURE 3:**
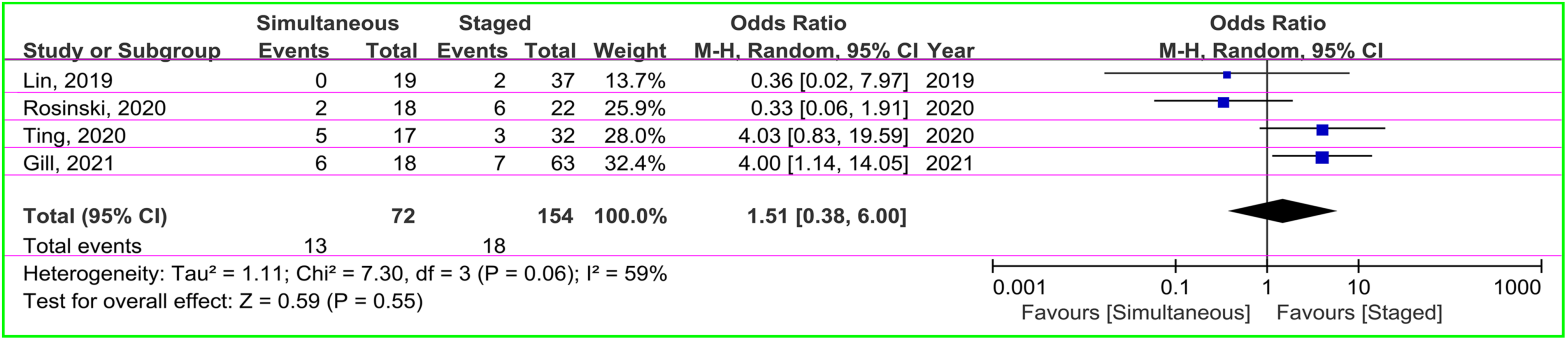
The forest plot displaying a comparison of the risk of reoperation between the two groups.

A total of 31 patients with reoperation were reported in four articles, including 13 (18.1%) in the simultaneous and 18 (11.7%) in the staged groups.^28^, ^32^, ^34^, ^35^ The OR was applied to estimate the incidence of reoperation. There was moderate heterogeneity (χ^2^ = 7.30, *I* ^2^ = 59%, *p* = 0.06), so the random‐effect model was used. The pooled OR was 1.51 (95% CI: 0.38–6.00, *p* = 0.55), suggesting statistically no significant in reoperation rate of patients in both groups (Figure 3).

### 3.5 Risk of shunt obstruction

**FIGURE 4:**
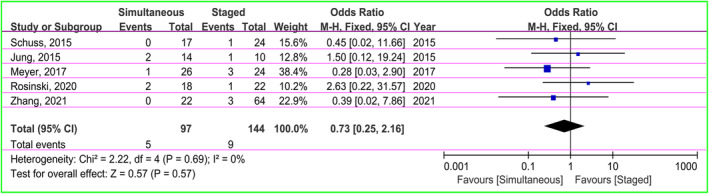
Forest plot of meta‐analysis on the occurrence of shunt obstruction risk in both groups.

There were five studies presenting data of shunt obstruction (*n* = 5 in the simultaneous groups; *n* = 9 in the staged groups).^29^, ^31^, ^33^, ^35^, ^37^ The OR as the effect size was used. There was no heterogeneity between groups (χ^2^ = 2.22, *I* ^2^ = 0%, *p* = 0.69), so the fixed‐effect model was applied. The pooled result indicated no significant difference in risk of shunt obstruction between groups (OR = 0.73, 95% CI: 0.25–2.16, *p* = 0.57; Figure 4).

### 3.6 Risk of hematoma

**FIGURE 5:**
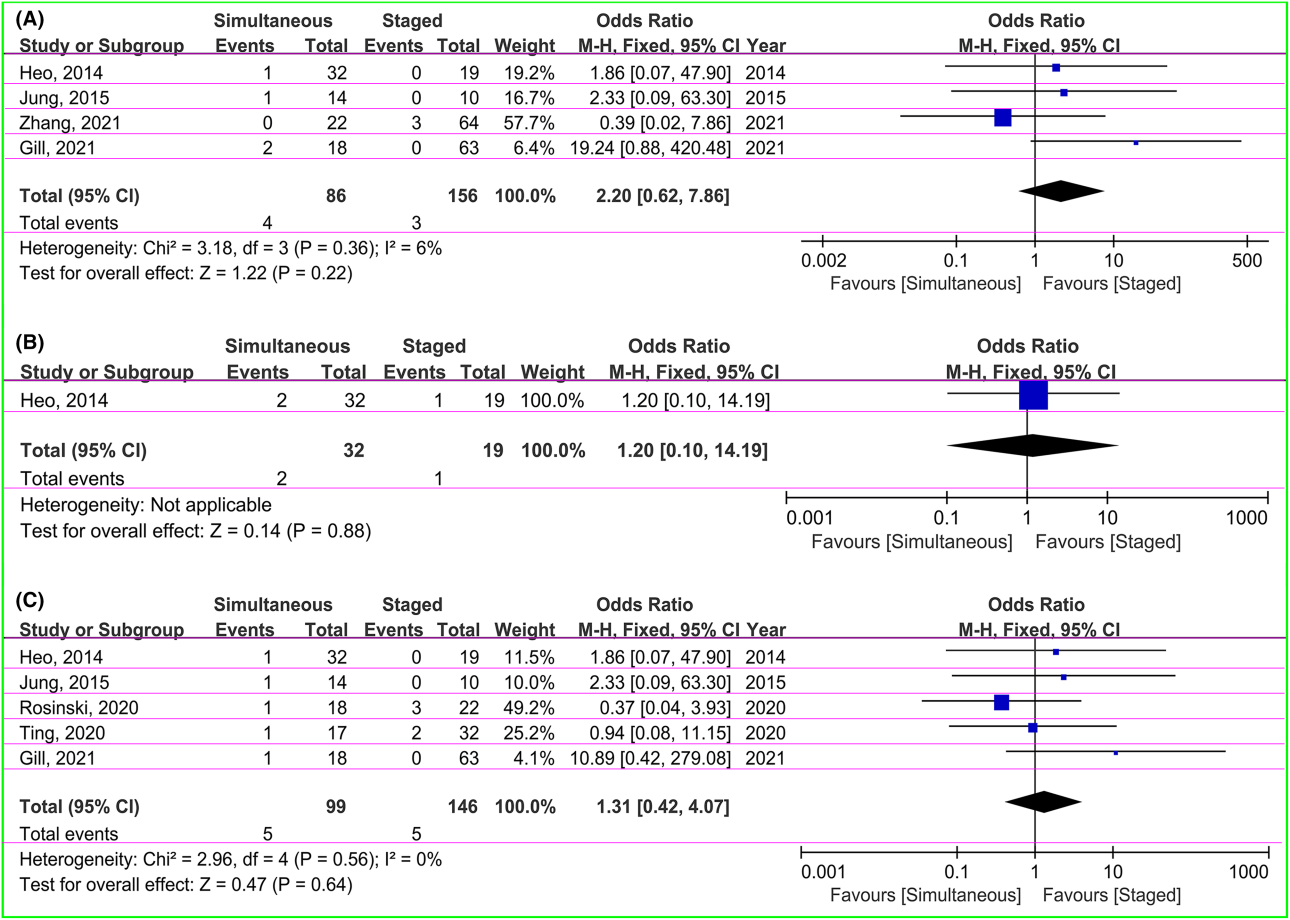
Forest plot indicating comparison of epidural hematoma (A), subdural hematoma (B), and intracranial hematoma (C) in simultaneous and staged groups.

The data of epidural hematoma, subdural hematoma, and intracranial hematoma were included in the pooled analysis. The seven patients in four studies had epidural hematoma, including 4 (4.7%) in the simultaneous groups and 3 (1.9%) in the staged groups.^28^, ^29^, ^30^, ^33^ Pooled finding with low heterogeneity (χ^2^ = 3.18, *I* ^2^ = 6%, *p* = 0.36) using fixed‐effect model did not suggest any statistically significant difference (OR = 2.20, 95% CI: 0.62–7.86, *p* = 0.22; Figure 5A). There was one article reporting three patients with subdural hematoma, including 2 (6.3%) in the simultaneous and 1 (5.3%) in the staged groups. The pooled OR was 1.20 (95% CI: 0.10–14.19, *p* = 0.88), indicating no statistically significant difference between two groups (Figure 5B).^30^ The five papers showed data of intracranial hematoma (five in the simultaneous and staged groups, respectively).^28^, ^30^, ^32^, ^33^, ^35^ The result of pooled analysis with no heterogeneity (χ^2^ = 2.96, *I* ^2^ = 0%, *p* = 0.56) using fixed‐effect model did not demonstrate any statistically significant difference in both groups (OR = 1.31, 95% CI: 0.42–4.07, *p* = 0.64; Figure 5C).

### 3.7 Risk of subdural effusion

**FIGURE 6:**
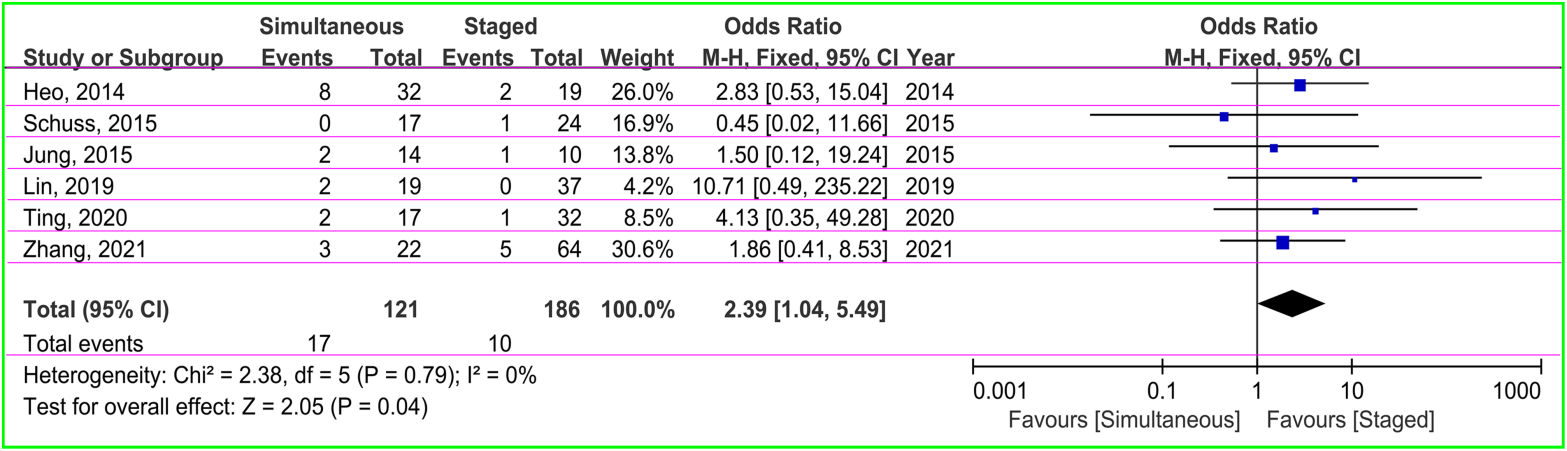
Forest plot of subdural effusion risk between two groups.

There were 27 patients suffering from subdural effusion in six studies, including 17 (14.0%) in the simultaneous and 10 (5.4%) in the staged groups.^29^, ^30^, ^31^, ^32^, ^33^, ^34^ The OR was used to estimate the incidence of subdural effusion between two groups. The heterogeneity test indicated no difference between studies (χ^2^ = 2.38, *I* ^2^ = 0%, *p* = 0.79), so the fixed‐effect model was applied. The pooled OR was 2.39 (95% CI: 1.04–5.49, *p* = 0.04), indicating the incidence of subdural effusion in the staged groups was statistically significantly lower compared with the simultaneous groups (Figure 6).

### 3.8 Subgroup analysis

Patients in the staged groups were divided into four subgroups: *CP after VPS*, *VPS after CP*, *both*, and *unknown*. They were compared with the simultaneous group respectively to explore the influence of the order of CP and VPS on risk of overall infection, central nervous system infection, cranioplasty infection, shunt infection, reoperation, shunt obstruction, epidural hematoma, intracranial hematoma, and subdural effusion. Some trials did not distinguish surgical procedures were defined as “both”. Studies that did not report a specific surgical order were defined as “unknown”. The findings of the subgroup analysis are shown in Table 3. The results demonstrated that the influences of the order of CP and VPS in prognosis are still not known.

**TABLE 3:** Subgroup analysis of staged procedures (both, CP after VPS, CP after VPS, and unknown).

Subgroups	Studies	χ^2^	*I* ^2^, %	Odds ratio	95%CI	*p* value
Overall infection	10	20.25	56	1.92	0.74–4.97	0.18
Both	4	5.62	47	1.51	0.41–5.58	0.54
CP after VPS	3	11.66	83	3.32	0.16–69.23	0.44
VPS after CP	2	2.44	59	1.87	0.18–19.29	0.60
Unknown	1	‐	‐	4.15	0.46–37.51	0.20
Central nervous system infection	6	5.34	6	1.50	0.68–3.31	0.31
Both	3	1.66	0	1.89	0.58–6.22	0.29
CP after VPS	2	2.65	62	1.72	0.15–19.19	0.66
VPS after CP	1	‐	‐	6.92	0.66–72.95	0.11
Unknown	0	‐	‐	‐	‐	‐
Cranioplasty infection	4	0.08	0	1.58	0.50–5.00	0.44
Both	2	0.03	0	1.60	0.39–6.53	0.51
CP after VPS	2	0.05	0	1.54	0.21–11.18	0.67
VPS after CP	0	‐	‐	‐	‐	‐
Unknown	0	‐	‐	‐	‐	‐
Shunt infection	3	2.63	24	1.30	0.38–4.52	0.67
Both	2	0.01	0	0.61	0.12–3.04	0.54
CP after VPS	1	‐	‐	10.48	0.47–231.80	0.14
VPS after CP	0	‐	‐	‐	‐	‐
Unknown	0	‐	‐	‐	‐	‐
Reoperation	4	1.11	59	1.51	0.38–6.00	0.55
Both	2	2.08	52	1.82	0.19–17.41	0.60
CP after VPS	2	4.32	77	1.19	0.10–13.77	0.89
VPS after CP	0	‐	‐	‐	‐	‐
Unknown	0	‐	‐	‐	‐	‐
Obstruction	5	2.22	0	0.73	0.25–2.16	0.57
Both	2	0.03	0	0.32	0.05–2.07	0.23
CP after VPS	2	0.72	0	1.31	0.21–8.04	0.77
VPS after CP	1	‐	‐	1.50	0.12–19.24	0.76
Unknown	0	‐	‐	‐	‐	‐
Epidural hemorrhage	4	3.18	6	2.20	0.62–7.86	0.22
Both	2	3.16	68	2.70	0.06–123.98	0.61
CP after VPS	0	‐	‐	‐	‐	‐
VPS after CP	1	‐	‐	2.33	0.09–63.30	0.61
Unknown	1	‐	‐	1.86	0.07–47.90	0.71
Intracranial hematoma	5	2.96	0	1.31	0.42–4.07	0.64
Both	1	‐	‐	10.89	0.42–279.08	0.15
CP after VPS	2	0.28	0	0.56	0.10–3.06	0.51
VPS after CP	1	‐	‐	2.33	0.09–63.30	0.61
Unknown	1	‐	‐	1.86	0.07–47.90	0.71
Subdural effusion	6	2.38	0	2.39	1.04–5.49	0.04
Both	2	1.08	7	2.96	0.83–10.56	0.09
CP after VPS	2	1.14	12	1.68	0.29–9.85	0.57
VPS after CP	1	‐	‐	1.50	0.12–19.24	0.76
Unknown	1	‐	‐	2.83	0.53–15.04	0.22

*Note*: *p* < 0.05 is favorable for staging.

Abbreviations: CI, confidence interval; CP, cranioplasty.VPS, ventriculoperitoneal shunt.

### 3.9 Sensitivity analysis and publication bias

**FIGURE 7:**
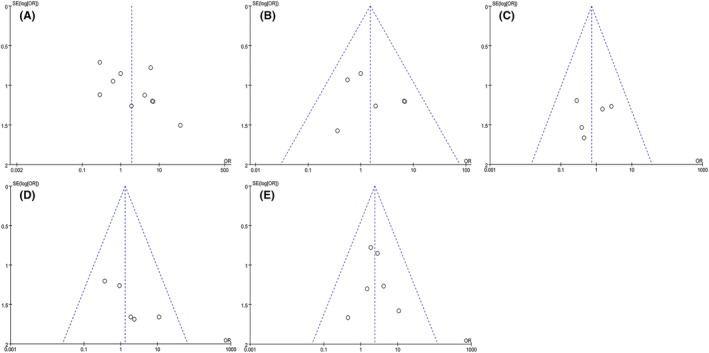
The funnel plots of overall infection (A), central nervous system infection (B), shunt obstruction (C), intracranial hematoma (D), and subdural effusion (E).

Sensitivity analysis was performed by individually removing each study from overall pooled analysis to evaluate the stability of findings. The results of this meta‐analysis were basically stable. However, individual studies can change the pooled result of subdural effusion. This could be associated with the small sample size. Funnel plots suggested that there was no significant publication bias for overall infection, central nervous system infection, shunt obstruction, intracranial hematoma, and subdural effusion (Figure 7).

## 4 DISCUSSION

Decompressive craniectomy is a common treatment for patients with refractory intracranial hypertension, and patients who have received DC some require CP and VPS for further treatment. However, due to the lack of guidelines or expert consensus, the order of performing CP and VPS often depends on the neurosurgeon's personal preference. In recent years, several studies have been conducted to evaluate the effect of simultaneous and staged CP and VPS on postoperative complication rates, reporting different perspectives.^28^, ^29^, ^30^, ^31^, ^32^, ^33^, ^34^, ^35^, ^36^, ^37^ However, there were characteristics such as small sample size and lack of unified conclusions. Therefore, we performed this meta‐analysis by pooling data from related studies to assess the effect of simultaneous and staged (also including the exploration of the order of staged) CP and VPS on postoperative infection, reoperation, shunt obstruction, hematoma, and subdural effusion.

Infection is one of the most observed complication after CP and VPS. In our meta‐analysis, there was no statistically significant difference in the incidence of postoperative infection. However, there was a higher trend in the simultaneous groups. On the one hand, in the simultaneous groups, longer operative duration and greater operative scope are risk factors for infection. Meyer et al.^37^ reported a higher infection rate in the cohort of patients and emphasized the need for more careful aseptic technique when performing multiple simultaneous surgeries. On the other hand, simultaneous surgery could be susceptible to infections caused by the materials used, such as cranial material and shunt devices.^28^ In addition, implanting two types of medical materials at the same time also puts patients at a higher risk of infection. This could be related to the microorganisms that colonize the surface of heterogeneous materials can cause recurrent infections.^42^ Study by Tsang et al.^43^ suggested that CP could theoretically increase the risk of infection in CSF shunts through direct contamination or hematogenous spread.^43^ In fact, the two influence and promote each other, other studies have shown that CSF shunt‐related infections can cause post‐CP infections through CSF circulation.^42^, ^44^ In addition, study from Schuss et al.^31^ indicated that poorer general and neurological conditions of patients might predispose neurosurgeons to perform simultaneous surgery to avoid possible complications from secondary surgery, which may also be a contributing factor to higher postoperative infection rates. Some researchers believed that using an antibiotic‐impregnated shunt system during surgery may reduce the incidence of infection.^45^, ^46^ However, there is an urgent need for large well‐designed randomized controlled trials to clarify the immediate and long‐term effect.

In this meta‐analysis, there were no statistically significant differences in the incidence of reoperation and shunt obstruction after CP and VPS between the simultaneous and staged groups. Several reasons can cause shunt obstruction.^47^ Although controversial, neurosurgery generally agrees that pathologically high levels of CSF protein and cells are associated with shunt obstruction.^48^, ^49^, ^50^ In addition, shunt siphoning resulting from overdrainage may cause shunts obstructed by a ventricular tissue protrusion.^51^ Causes of reoperation include infection, shunt dysfunction, bone flap resorption, and cosmetic defects, etc.^29^ Clinical study demonstrated that the presence of shunts was an independent risk factor for bone flap resorption after CP.^52^ Shunt placement reduces dura mater adhesion to the bone flap, ICP fluctuations, and negatively affects cranial repair, thereby contributing to bone flap resorption.^53^, ^54^, ^55^, ^56^, ^57^ However, Behbahani et al.^58^ reported that the presence of shunts had a protective effect on bone flap resorption. Therefore, further studies are needed to determine the relationship between bone flap absorption and the presence of shunts.

In addition, different skull materials may also involve different risks of pressure absorption of skin flaps at ultra‐long‐term, which could occur in patients with both artificial skull and VPS device. This condition is not affected by the surgical plan, but mainly due to the artificial skull itself. It is worth noting that ultra‐long‐term complications are mostly caused by artificial materials with meshes or sharp edges. First, sharp edges are due to the blunting treatment technology of the material edge itself, and second to the deformation of the material itself. Once the material slowly changes from convex to concave due to the multidimensional pressure difference, its edge is forced to curl up, and the scalp is gradually consumed under shear force. Therefore, in the selection of artificial skull, we need to consider the structure of the material itself and its mechanical effects (long‐term material deformation risk). As of now, there are advantages and disadvantages to different artificial skull materials, and further long‐term follow‐up studies on various materials are worthwhile. Therefore, only by fully understanding the material characteristics and combining them with different surgical techniques could reduce the above‐mentioned long‐term complications to some extent. For example, clear understanding of the scalp anatomy, sufficient and thorough skin flap thickness, and clever combination with the mechanical properties of the connecting material.

There was no statistical difference in the incidence of hematoma after CP and VPS between two groups. However, the simultaneous groups was significantly associated with a higher incidence of subdural effusion. Complications such as hematoma and subdural effusion in patients undergoing simultaneous CP and VPS have been reported to be associated with VPS‐induced sunken down effect of the brain.^30^, ^33^ Heo et al.^30^ suggested a higher incidence of hematoma and subdural effusion in the simultaneous groups and indicated that this might be due to the difficulty in regulating VPS pressure in these patients. Therefore, it is particularly concerning to reasonably match the type of shunt. Fixed pressure shunt with limited adjustable pressure range, making precise adjustments on an individual basis difficult. However, programmable shunts can set the appropriate shunt pressure based on the patient's CSF pressure. Of course, its advantage lies in this, it could adjust the pressure in time after the operation and reduce the complications caused by insufficient or excessive shunt.

Furthermore, difficult‐to‐eliminate epidural dead space may increase the risk of hematoma and subdural effusion.^59^ Study by Liao and Kao^60^ further confirms the above theoretical analysis and demonstrated that temporary clipping of the shunt device could eliminate the dead space between the skull plate and the dura, thereby reducing the risk of subdural effusion and hematoma. Similar results reported from Jung et al, arguing that blocking CSF drainage could avoid the sunken down effect of the brain and prevent the occurrence of hematoma and subdural effusion.^33^, ^61^ Given this, it is necessary to choose appropriate flow rate and volume of CSF shunt base on programmable shunts. If these complications are not properly avoided, the risk of reoperation may be increased. However, the precise regulation of CSF in time needs to be further explored. In addition, the guidelines for regulation also need to be clarified and refined. Obviously, there is no uniform measure of whether the VPS pressure in these patients is appropriate. Just imagine, the invention of a system that intelligently calculates flow rate based on ideal indicators may have potential value of clinical application. But before that, we need to clarify what is the ideal indicators.

In subgroup analysis, there were no statistical differences in all comparisons. There is no consensus on the sequence of CP and VPS in staged surgery. The traditional clinical experience is usually CP after VPS. But, a one‐size‐fits‐all approach may be arbitrary. Study by Oh et al.^62^ confirmed this and their found that the outcomes of VPS after CP tend to be better than CP after shunt operation in patients with large, concave flaccid skull defect. If the VPS is performed first, the patient's ICP will be reduced, resulting in an increase in the difference between atmospheric pressure and ICP, thereby disrupting the normal anatomy of the brain, blood supply, and CSF circulation, which may cause SSFS with symptoms including epilepsy, headaches, dizziness, language deficits, motor deficits, or even paradoxical herniation.^63^, ^64^, ^65^ In contrast, initial CP can significantly improve DC‐induced CSF dynamics dysfunction, thereby partially relieving symptoms of hydrocephalus and potential avoiding the need for subsequent shunting.^66^, ^67^ But it is not absolute, Morton et al.^68^ found that post‐CP was associated with a 9.0% risk of hydrocephalus. In addition, for patients with tense convex cranial defects, VPS must be performed first, and CP should be performed after the bone window is slightly sunken or flattened.^69^ However, as with the surgical options, the diagnosis of hydrocephalus in patients with cranial defects is also highly challenging. The cerebrospinal fluid tap test has been regarded as a core tool for the prediction of VPS effectiveness in hydrocephalus patients. Multiple cerebrospinal fluid tap test could be put on the agenda when it is difficult to make a choice through intuitive clinical manifestations. Combined with the above findings, we cautiously believe that staged (also its order) or simultaneous surgery should be selected according to the specific conditions of the patients.

### 4.1 Limitations

This meta‐analysis has some limitations. The 10 included studies were all single‐center retrospective studies with heterogeneity of study protocols. There may be some relevant articles that were missed, even though we searched major databases and tracked the reference lists of included studies. In addition, there were differences in the CP transplantation materials, the degree of brain bulging, timing of surgery following the initial causative event, and the interval and sequence between CP and VPS in different studies. However, it will not be possible to answer these issues unambiguously, based on data that are difficult to subdivide into subgroups. Thus, the above questions could be the focus of future study. Although 10 studies were included in the pooled analysis, the total sample size was relatively small. Our findings should be analyzed with caution, and neurosurgeons in practice should design individualized treatment plans based on each patient's condition. Prospective studies and randomized controlled trials with strict inclusion criteria are needed in the future to further analyze the pros and cons of simultaneous and staged CP and VPS. Furthermore, neurological functional outcome should also be investigated.

## 5 CONCLUSIONS

The differential diagnosis cannot be ignored between encephalocele and hydrocephalus. Once the diagnosis of hydrocephalus combined with a cranial defect is confirmed, follow‐up issues will be on the agenda. While these findings were not stable, the authors cautiously indicated that patients with cranial defects and hydrocephalus who undergo staged CP and VPS procedures might benefit from a potentially lower trend in complication rate. However, more studies, ideally using larger, prospective, and multicentre designs are needed to clarify this. Ultimately, neurosurgeons in practice should make individualized decisions based on each patient's condition (also including cerebrospinal fluid tap test), to guide the formulation of staged or imultaneous surgical protocols, even the order of CP and VPS in staged surgeries.

## FUNDING INFORMATION

The funding was provided by the National Natural Science Foundation of China (NSFC Grants 82171382, 81870968, and 81901243).

## CONFLICT OF INTEREST STATEMENT

The authors report no conflicts of interest in this work.

## INFORMED CONSENT

N/A.

## Data Availability

The data that support the findings of our study could be available from contacting the corresponding author.
